# Interplay of Calcium and Nitric Oxide in improvement of Growth and Arsenic-induced Toxicity in Mustard Seedlings

**DOI:** 10.1038/s41598-020-62831-0

**Published:** 2020-04-23

**Authors:** Rachana Singh, Parul Parihar, Sheo Mohan Prasad

**Affiliations:** 1 Ranjan Plant Physiology and Biochemistry Laboratory, Department of Botany, University of Allahabad, Allahabad, U.P. India; 2 School of Bioengineering and Biosciences, Lovely Professional University, Phagwara, India

**Keywords:** Plant sciences, Plant physiology

## Abstract

In this study, Ca^2+^ mediated NO signalling was studied in response to metalloid (As) stress in *Brassica* seedlings. Arsenic toxicity strongly suppressed the growth (fresh weight, root and shoot length), photosynthetic pigments, Chl *a* fluorescence indices (Kinetic traits: *F*_*v*_, *F*_*m*_, *F*_*v*_*/F*_*o*_, *F*_*m*_*/F*_*o*_, *ФP*_*o*_ or *F*_*v*_*/F*_*m*_, *Ψ*_*o*_, *ФE*_*o*_, *PI*_*ABS*_, *Area* and *N* and redox status (AsA/DHA and GSH/GSSG ratios) of the cell; whereas energy flux traits: *ABS/RC*, *TR*_*o*_*/RC*, *ET*_*o*_*/RC* and *DI*_*o*_*/RC* along with *F*_*o*_, *F*_*o*_*/F*_*v*_, *F*_*o*_*/F*_*m*_, *ФD*_*o*_ and *S*_*m*_) were enhanced. Further, addition of EGTA (Ca^2+^ scavenger) and LaCl_3_ (plasma membrane Ca^2+^ channel blocker) to As + Ca; while c‒PTIO (NO scavenger) and l‒NAME (NO synthase inhibitor) to As + SNP treated seedlings, siezed recovery on above parameters caused due to Ca^2+^ and NO supplementation, respectively to As stressed seedlings thereby indicating their signalling behaviour. Further, to investigate the link between Ca^2+^ and NO, when c‒PTIO and l‒NAME individually as well as in combination were supplemented to As + Ca treated seedlings; a sharp inhibition in above mentioned traits was observed even in presence of Ca^2+^, thereby signifying that NO plays crucial role in Ca^2+^ mediated signalling. In addition, As accumulation, ROS and their indices, antioxidant system, NO accumulation and thiol compounds were also studied that showed varied results.

## Introduction

Arsenic (As) exposure has become a major threat for world agriculture that causes adverse effect on crop productivity by inhibiting cell functioning. The metalloid (As) mainly arises from geothermal weathering of rocks and human activities^1^. The toxicity of As depends on As species. Among the two inorganic species, arsenite (As^III^), which prevails in anaerobic environment, enters in plant system through aquaporin channels with greater affinity for thiol groups; while arsenate (As^V^) prevalent in aerobic condition/ soil, being analogue of inorganic phosphate (iP), enters *via* inorganic phosphate transporters, competes with phosphate and replaces phosphate from ATP thereby affecting the energy metabolism of cell^2,3^. Arsenic accumulation causes growth suppression which involves many biochemical and physiological changes in plants including oxidative stress, injuries to membrane and thereby affecting redox state of the cell^4–6^, which is detrimental for plant survival under As toxicity. Additionally, As toxicity negatively regulates chlorophyll (Chl) biosynthesis, PS II photochemistry and ribulose 1,5‒bisphosphate carboxylase/ oxygenase (RuBisCO) activity, thereby inhibiting photosynthetic efficiency^6–9^. Previous studies have explicated the prominence of upholding a favourable antioxidants level and redox status of cell to encounter the damage caused due to metalloid exposure^4–6^.

Calcium (Ca^2+^) is a pervasive and pivotal secondary messenger in signal transduction network^10^ under both stressed and non‒stressed situations. In several studies, it has been shown that Ca^2+^ is involved in regulation of plant responses such as photosynthetic electron transport rate, enzyme activities of Calvin cycle and activities of key antioxidant enzymes^5,11,12^, stabilizing the structural integrity of membranes by making bond with phospholipid bilayer^13^ under various environmental stresses including As. Nitric Oxide (NO), on the other hand is a bioactive gaseous free radical and also as inter and intracellular signalling molecule, and regulates numerous biochemical, physiological and molecular processes in plants under variable conditions^4,14,15^. Peto *et al*.^16^ reported that NO application encounters excessive ROS production by metal/ metalloids in two ways: either acting as a free radical reacting with ROS to neutralize them or as a signalling molecule initiating gene expression in molecular cascade. In proteins, NO induced post‒translational modification is carried out by nitrosylation of their cysteine residue. Wu *et al*.^17^ reported that NO application improves photosynthetic rate by (i) channelizing excess energy by increasing carotenoid or other antenna molecules and (ii) increasing quantum yield of PS II, under salinity stress in *Solanum melongena* seedlings.

From the available literature, it is clear that both Ca^2+^ and NO plays multiple roles in regulating key physiological processes in stressed as well as non‒stressed situations; however, their cumulative effect in orchestrating plant responses to different environmental cues have not been well established. Therefore, future studies are needed to understand the intensive interaction and interrelation of Ca^2+^ and NO in various physiological, histochemical and metabolic approaches suffering from arsenic toxicity in plants. Towards this objective, the key components *i.e*. growth and growth regulating parameters: Chl *a* fluorescence, redox status of cell, enzymatic and non‒enzymatic antioxidants and levels of thiol compounds were assessed in the present investigation.

## Results and discussion

### Ca^2+^ and NO recover As‒induced damage in phenotypic appearance

**Figure 1:**
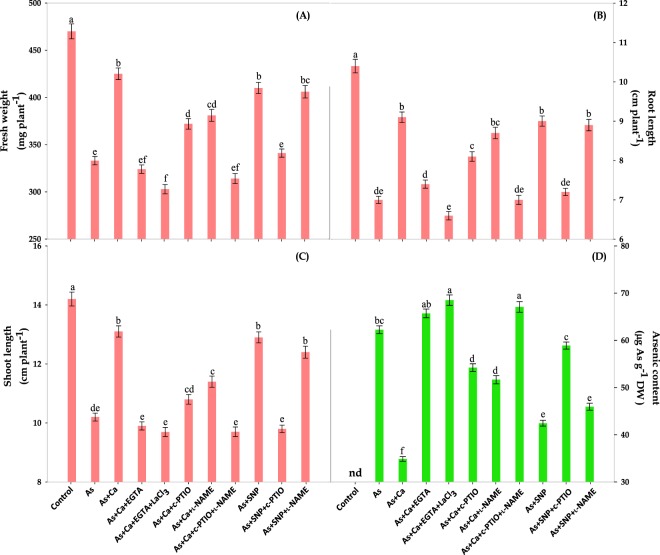
Effect of CaCl_2_(Ca_2+_) and SNP (NO) on the phenotypic appearance: (**A**) fresh weight, (**B**) root length, (**C**) shoot length and (**D**) As content of As‒challenged *Brassica* seedlings subjected to different modulators (**EGTA:** ethylene glycol‒bis(2‒aminoethylether)‒*N,N,N*′*,N*′‒tetraacetic acid, a Ca scavenger, **LaCl**_**3**_**:** lanthanum chloride, a plasma membrane Ca channel blocker, **c‒PTIO:** 2‒4‒carboxyphenyl‒*4,4,5,5*‒tetramethylimidazoline‒1‒oxyl‒3‒oxide, a NO scavenger and _**L**_**‒NAME:** N^ω^‒nitro‒L‒arginine methyl ester hydrochloride, a nitric oxide synthase inhibitor). Data signifies the mean ± standard error of five replicates. Bars followed by different letters are significantly different at *p* < *0.05* level according to Tukey test. Where ‘nd’ is ‘no detection’ of As.

**Figure 2:**
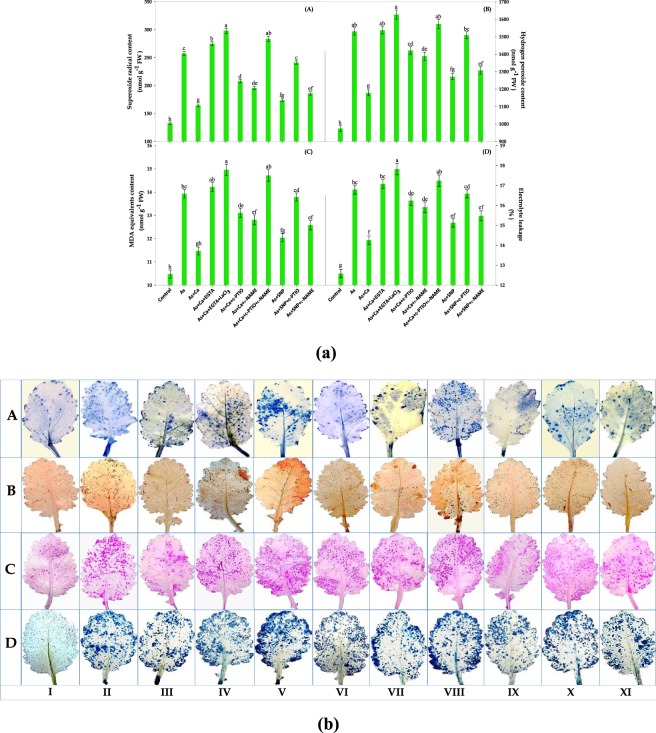
(**a**) Effect of CaCl_2_ and SNP on the contents: (A) super oxide radical (SOR), (B) hydrogen peroxide (H_2_O_2_), (C) malondialdehyde (MDA) equivalents and (D) electrolyte leakage of the leaves of As‒challenged *Brassica* seedlings subjected to different modulators. Data signifies the mean ± standard error of five replicates. Bars followed by different letters are significantly different at *p* < *0.05* level according to Tukey test. (**b**) Histochemical detection of (A) SOR, (B) H_2_O_2_ and (C) lipid peroxidation (MDA equivalents) and (D) loss of membrane integrity (electrolyte leakage) showing the effect of CaCl_2_ and SNP on the leaves of As‒challenged *Brassica* seedlings subjected to different modulators. [(I) Control, (II) As, (III) As + Ca, (IV) As + Ca + EGTA, (V) As + Ca + EGTA + LaCl_3_, (VI) As + Ca + c‒PTIO, (VII) As + Ca + l‒NAME, (VIII) As + Ca + c‒PTIO + l‒NAME, (IX) As + SNP, (X) As + SNP + c‒PTIO and (XI) As + SNP + l‒NAME].

**Figure 3:**
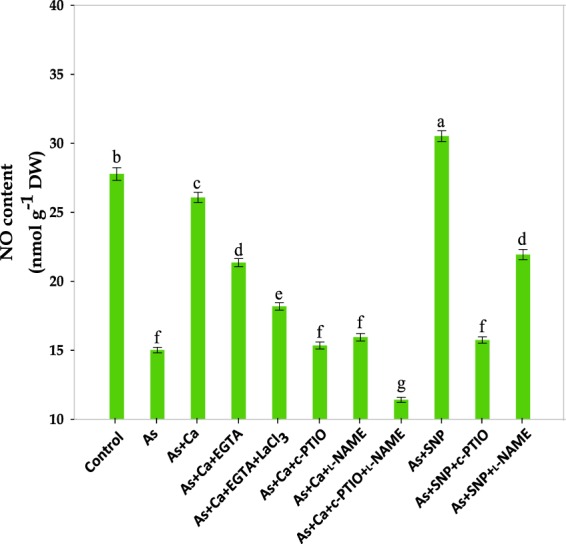
Effect of different modulators of Ca^2+^ and NO on NO content of the leaves of As‒challenged *Brassica* seedlings. Data signifies the mean ± standard error of five replicates. Bars followed by different letters are significantly different at *p* < *0.05* level according to Tukey test.

To examine the Ca^2+^ mediated NO signalling in alleviating As‒induced toxicity, *Brassica* L. seedlings were treated with different donors, scavengers and inhibitors of Ca^2+^ and NO. As expected, metalloid (As) stress caused deteriorating effect on growth and declined the fresh weight, root and shoot length by 29, 33 and 28%, respectively of test seedlings (Fig. 1), which is manifested by increased reactive oxygen species (ROS) production that caused lipid peroxidation, protein oxidation and loss to membrane integrity leading to electrolyte leakage (Figs. 2a,b). However, under As stress, both CaCl_2_ and SNP treatment counteracted As‒induced negative impact on FW, RL and SL of test seedlings (Figs. 1a‒c), which agree with the greater accumulation of NO than As‒stressed seedlings alone (Fig. 3). The Ca^2+^ and NO induced positive response on growth have also been reported by Singh *et al*.^5^ and Siddiqui *et al*.^15^ in mustard and tomato seedlings, respectively. Further, a significant inhibition of growth after EGTA and LaCl_3_ treatment indicated that both of them arrests Ca^2+^‒induced positive impact on growth agreeing the fact of involvement of Ca^2+^ as signalling molecule. Knight *et al*.^11^ have also reported that lanthanum (La) and EGTA inhibit the salt‒and mannitol‒induced (Ca^2+^)_cyt_ elevations in *Arabidopsis*. In a previous study, Xu *et al*.^18^ have reported that ABA protects tall fescue plant from oxidative injuries by promoting NO release (*via* activating NOS) thereby triggering the activities of antioxidant enzymes. Therefore, also in order to study the possible link between Ca^2+^ and NO signalling, As + Ca treated *Brassica* seedlings were treated with NO scavenger: c‒PTIO and synthase inhibitor: l‒NAME. Interestingly, the growth of As + Ca treated seedlings was abolished in presence of c‒PTIO and l‒NAME (Figs. 1a‒c), suggesting that NO is required for the maximal and sustained signalling of Ca^2+^, which corresponds to reduced NO accumulation in presence of c‒PTIO and l‒NAME (Fig. 3). Lanteri *et al*.^19^ also reported that NO is required for the maximal activity of Ca^2+^‒dependent protein kinase (CDPK) for adventitious root formation in *Cucumis sativus*. Results of As accumulation suggests that both CaCl_2_ and SNP counteracted the As accumulation in test seedlings; however in presence of c‒PTIO and/or l‒NAME even Ca^2+^ was unable in restricting As accumulation, therefore higher As content was observed in c‒PTIO + l‒NAME treated seedlings than As‒stressed seedlings alone (Fig. 1d).

### Ca^2+^ and NO rescue As‒induced losses of Chls and Car and recovers photosynthetic rate and PS II photochemistry

To further elucidate the role of NO in Ca^2+^‒induced signalling on photosynthetic performance, photosynthetic pigments: Chls (*a* and *b*) and Car were examined. Arsenic induced reduction in the levels of Chl *a*, *b* and Car was in accordance with the reduced plant growth (Fig. 1), signifying that As impaired photosynthetic ability of plants (Table 1) by disrupting chloroplast structure and pigments’ biosynthesis^6^. Calcium and NO, on the other hand, rescued the As‒induced loss in photosynthetic pigments content that partly attributed to lowering of ROS, which might have prevented photo‒oxidative damage of photosynthetic apparatus^14,15^. However, As‒induced damage was severe when As + Ca treated seedlings were supplemented either with EGTA + LaCl_3_ or c‒PTIO + l‒NAME, which corresponds to the decreased values of photosynthetic rate (Table 1).

**Table 1:** Effect of CaCl_2_ and SNP on the levels of photosynthetic pigments content: chlorophyll (Chl) *a*, Chl *b* and carotenoids (Car) and photosynthetic performance of As‒challenged *Brassica* seedlings subjected to different modulators.

Treatments	Pigments content (µg mg^−1^ FW)			Photosynthesis (µmol O_2_ evolved (g FW)^−1^ h^−1^)
	Chl *a*	Chl *b*	Car	
Control	1.285 ± 0.020^a^	0.428 ± 0.006^a^	0.313 ± 0.005^a^	32.17 ± 0.53^a^
As	1.111 ± 0.016^cd^	0.312 ± 0.004^d^	0.282 ± 0.004^cd^	18.72 ± 0.24^g^
As + Ca	1.187 ± 0.018^b^	0.361 ± 0.006^bc^	0.293 ± 0.005^ab^	30.25 ± 0.43^b^
As + Ca + EGTA	1.106 ± 0.014^cd^	0.311 ± 0.004^d^	0.274 ± 0.004^cd^	16.15 ± 0.22^h^
As + Ca + EGTA + LaCl_3_	1.031 ± 0.016^d^	0.301 ± 0.004^d^	0.262 ± 0.003^d^	12.04 ± 0.19^i^
As + Ca + c‒PTIO	1.147 ± 0.018^bc^	0.343 ± 0.005^c^	0.284 ± 0.004^bc^	22.64 ± 0.34^ef^
As + Ca + l‒NAME	1.164 ± 0.020^b^	0.350 + ± 0.006^bc^	0.284 + ± 0.004^bc^	24.05 ± 0.40^de^
As + Ca + c-PTIO + l‒NAME	1.070 ± 0.016^cd^	0.293 ± 0.004^d^	0.264 ± 0.003^cd^	15.89 ± 0.26^h^
As + SNP	1.179 ± 0.017^b^	0.363 ± 0.006^bc^	0.290 ± 0.005^b^	27.42 ± 0.39^c^
As + SNP + c‒PTIO	1.133 ± 0.015^bc^	0.374 ± 0.006^b^	0.276 ± 0.003 ^cd^	21.01 ± 0.26^f^
As + SNP + l‒NAME	1.169 ± 0.017^b^	0.359 ± 0.004^bc^	0.291 ± 0.005^b^	25.81 ± 0.41^cd^

Data signifies the mean ± standard error of five replicates. Values within same column followed by different subscripts are significantly different at *p* < *0.05* level according to Tukey test.

**Figure 4:**
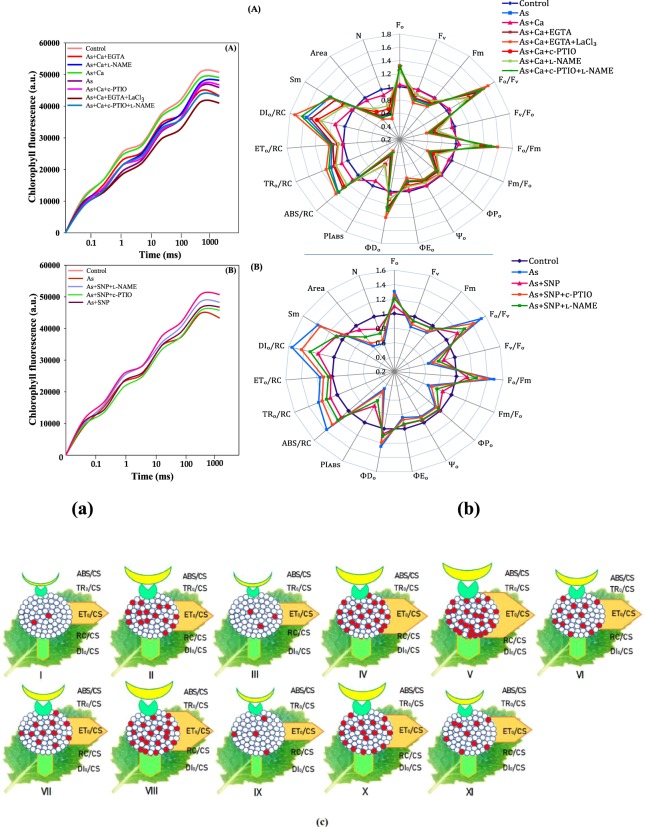
Effect of CaCl_2_ and SNP on the **(a)** chlorophyll *a* fluorescence OJIP transient curves and **(b)** spider plots for OJIP parameters inferred from chlorophyll *a* fluorescence OJIP transient in the leaves of As‒challenged *Brassica* seedlings subjected to different modulators. Data signifies the mean ± standard error of five replicates. Bars followed by different letters are significantly different at *p* < *0.05* level according to Tukey test. (**c**). The leaf model representing phenomenological energy fluxes per excited cross section (CS) in the leaves of As‒challenged *Brassica* seedlings subjected to different modulators. [(I) Control, (II) As, (III) As + Ca, (IV) As + Ca + EGTA, (V) As + Ca + EGTA + LaCl_3_, (VI) As + Ca + c‒PTIO, (VII) As + Ca + _L_‒NAME, (VIII) As + Ca + c‒PTIO + _L_‒NAME, (IX) As + SNP, (X) As + SNP + c‒PTIO and (XI) As + SNP + _L_‒NAME]. The relative value for the measured parameters is the mean of quintuplicates (n = 5). The width of arrow corresponds to the intensity of flux parameters; ABS/CS: absorption flux per CS, TR_0_/CS: trapped energy flux per CS, ET_0_/CS, electron transport flux per CS and DI_0_/CS: dissipated energy flux per CS. Circles embedded in circle (RC/CS) are percentage of active/inactive RCs, where white circles are representing reduced Q_A_ RCs (active) and red circles non‒reducing Q_A_ RCs (inactive). RCs: reaction centres as described by Sitko *et al*.^62^.

Further, to reveal the Ca^2+^ mediated NO role in structural and functional properties of PS II, the OJIP transient curves as well as biophysical traits deduced from OJIP were studied. A sharp drop in O‒J, J‒I and I‒P transient curves, which denotes the sequential reduction of electron acceptor pool of PS II, indicates that PS II was the major target of As (Fig. 4a). The decline in O‒J‒I‒P transient curve could be attributed to damage at the donor side of PS II restricting the flow of electron between OEC and PS II^20–24^. Moreover, the drop in OJIP transient curves was intense upon c‒PTIO + l‒NAME supplementation to As + Ca treated seedlings indicating that in absence of NO, the effect of As became more severe (even in presence of Ca^2+^), which could be due to: (i) inhibition in electron transport rate on the donor side of PS II as reflected by decreased values for area over the fluorescence curve (*Area*), which consequently decreased the maximum quantum yield for primary photochemistry (*ФP*_*o*_) thereby leading to accumulation of P_680_^+^ (Fig. 4b)^17,25^ and (ii) decline in the size and number of active photosynthetic RCs (*F*_*v*_*/F*_*o*_), disrupting electron transfer beyond Q_A_^−^ thus, higher initial fluorescence (*F*_*o*_) was measured in presence of NO scavenger and NOS inhibitor. Accordingly, a certain suppression in the values for *Ψ*_*o*_ (that designates trapped exciton moves an electron into the ETC beyond Q_A_^−^) and the quantum yield of electron transport (*ФE*_*o*_) was detected (Fig. 4b), which consequently declined the pool size of Q_A_^−^ (acceptor side of the PS II^17,26,27^); suggesting lethargic flow of electrons from PS II to PS I and restriction of Q_A_^−^ reoxidation (Q_A_^−^‒Q_A_), which could be associated with poor diffusion of PQ across the thylakoid membranes^28^. Indeed, the higher *F*_*o*_*/F*_*m*_ designates that Q_A_ reduction rate was much higher than its reoxidation rate by Q_B_ and PS I activity under As + Ca + c‒PTIO + l‒NAME treatment. Increasing values for the quantum yield of energy dissipation (*ФD*_*o*_) and dissipated energy flux (*DI*_*o*_*/RC*) (Fig. 4b) under As + Ca + c‒PTIO + l‒NAME and As + Ca + EGTA + LaCl_3_ treatment, advocate that excess excitation energy was converted to thermal dissipation in order to maintain the energy balance between absorption and consumption, and thus minimize the potential of photo‒oxidative damage^8^. The decrease in *F*_*m*_*/F*_*o*_ parameter reflects the damaging effect of As on the structural integrity of the PS II RCs^29^. Further, the increased *S*_*m*_ (refers the pool of electron transporters between PS II and the acceptor side of PS I) value under As + Ca + c‒PTIO + l‒NAME and As + Ca + EGTA + LaCl_3_ implies that heterogeneity of PQ increased the electron donation capacity and Q_A_ reduction on acceptor side of PS II, suggesting that As along with c‒PTIO + l‒NAME and EGTA + LaCl_3_ decreased the total electron accepting capacity^30^.

The progressive drop in overall performance of PS II (*PI*_*ABS*_) upon NO scavenger and NOS inhibitors’ treatment could have resulted from the inactivity of RCs (*F*_*v*_*/F*_*o*_), and these RCs then changes into ‘energy sinks/heat sink’, that absorb light but were unable to store the excitation energy and dissipate total energy as heat/fluorescence as deduced by *ФP*_*o*_ (*F*_*v*_*/F*_*m*_), which consequently changes the average antenna size linked to each active RC (*ABS/RC*^14,31^). Therefore, *ABS/RC* was found to increase under above situation because the reduced number of active RCs favour for increasing the necessary numbers of RC turnovers for complete reduction of the PQ pool (*N*) (Fig. 4b). The *TR*_*o*_*/RC*, which refers only to active RCs (Q_A_‒Q_A_^−^), was increased suggesting that either (i) all the Q_A_ might have been reduced (Q_A_^−^) but were not able to oxidize back (Q_A_) or (ii) the reoxidation of Q_A_^−^ (Q_A_^−^‒Q_A_) was inhibited under Ca^2+^ and NO scavenger/synthase inhibitor treated As stressed seedlings, so that Q_A_ was unable to transfer electrons efficiently to Q_B_^31^. The *DIo/RC*, which reflects the ratio of the dissipation of untrapped excitation energy from all the RCs with respect to the number of active RCs, was increased (Fig. 4b) due to higher energy dissipation from the active RCs under As toxicity^14^. Furthermore, increased *F*_*o*_*/F*_*v*_ refers to damaging effects on OEC, which could be due to the decline in uptake of mineral nutrients like Mn, an important component of OEC^6^, as it suggested by Samborska *et al*.^24^ that mineral nutrient deficiency tends to affect the fluorescence parameters variably.

The leaf model for phenomenological energy fluxes showed that As toxicity caused an increase in absorption flux per CS (ABS/CS), trapped energy flux per CS (TR_0_/CS), electron transport flux per CS (ET_0_/CS) and dissipated energy flux per CS (DI_0_/CS) along with the number of inactive/closed RCs (RC/CS) (Fig. 4c).

Calcium and NO, on the other hand alleviated the negative impact of As by restoring the structural attributes of PS II as favoured by increased *ФP*_*o*_, *F*_*m*_*/F*_*o*_ and *F*_*v*_*/F*_*o*_ and decreased *F*_*o*_*/F*_*v*_ values. The positive role of Ca^2+^ on *ФP*_*o*_ may due to the mineral ion homeostasis as discussed by Ahmad *et al*.^13^ in tomato seedlings. Upon CaCl_2_ and SNP application, improvement in *F*_*v*_ parameter was strongly supported by a reduction in *F*_*o*_, which favoured imitation of the PS II acceptor side^30^. Furthermore, an improvement in the electron transport rate of the photosynthetic ETC was noticed as deduced from the high *F*_*v*_*/F*_*o*_ values (Fig. 4b). The restoration in *F*_*m*_ value upon CaCl_2_ and SNP application suggested that either they might have increased Mn ion and extrinsic proteins of OEC, which affected the electron donation from water to PS II^32^ or might have caused the conformational changes in D1 protein, thereby altering the properties of PS II electron acceptors^33^, which augmented PS II activity^6^. The NO might have improved the electron transport rate from OEC to D1 protein and gene expression belongs to core reaction center (Psb) of PSII complex such as *psbA*, *psbB* and *psbC* as argued by Chen *et al*.^14^ in heat‒stressed tall fescue leaves. Upon CaCl_2_ and SNP addition, a sharp drop in *ABS/RC*, *TR*_*o*_*/RC*, *ET*_*o*_*/RC* and *DI*_*o*_*/RC* (Fig. 4b) specify that PS II apparatus was able to tackle the balance of energy fluxes for absorption, trapping and transport of electrons through active PS II RCs under As toxicity^14,34^ thereby improving overall performance of PS II, as agreed with higher *PI*_*ABS*_ values, which can also be supported by improved *Area* value under similar conditions (Fig. 4b).

### Ca^2+^ and NO improve antioxidant defense system to counteract As‒induced oxidative stress and injuries

**Figure 5:**
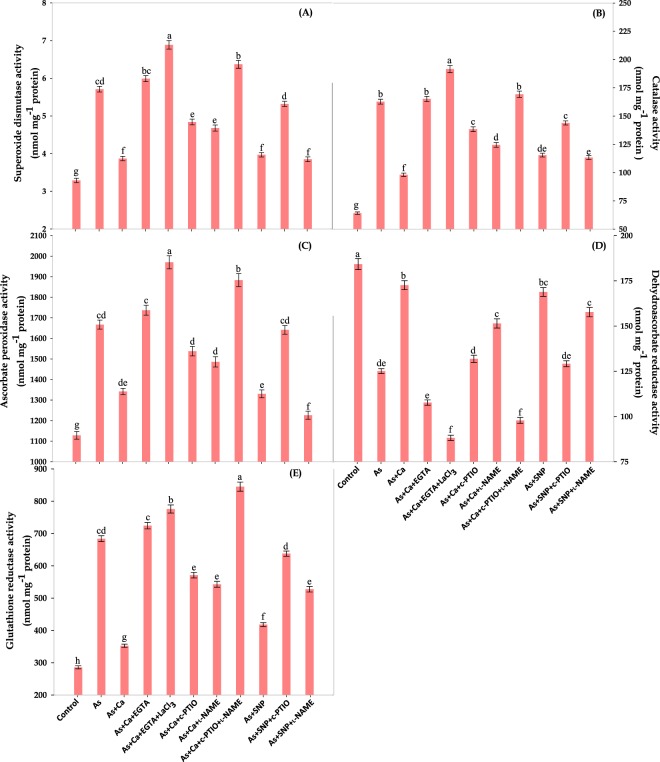
Effect of CaCl_2_ and SNP on the activities of enzymatic antioxidants: **(A)** superoxide dismutase (SOD), **(B)** catalase (CAT), **(C)** ascorbate peroxidase (APX)**, (D)** dehydroascorbate reductase (DHAR) and **(E)** glutathione reductase (GR) of the leaves of As‒challenged *Brassica* seedlings subjected to different modulators. Data signifies the mean ± standard error of five replicates. Bars followed by different letters are significantly different at *p* < *0.05* level according to Tukey test.

In our study, the excess accumulation of As in leaf tissues of *Brassica* seedlings exhibited severe oxidative stress as evident by enhanced ROS: O_2_˙^−^ and H_2_O_2_ levels (Fig. 2). The As‒induced ROS production caused oxidative injuries by peroxidizing lipid membranes together with loss of membrane integrity which were correlated with significant increase in electrolyte leakage and MDA equivalents levels (Figs. 2a‒b). Further, CaCl_2_ and SNP addition counteracted the As‒induced loss in cell structure and function by decreasing ROS and the indices of damage as evident by increased NO and decreased As content (Figs. 1d and 3). The increased NO might have formed a less toxic peroxynitrite (ONOO^−^)^16,35^ or induced various ROS‒scavenging enzyme activities like superoxide dismutase (SOD), catalase (CAT) and ascorbate peroxidase (APX) (Figs. 5a‒c) as was discussed by Siddiqui *et al*.^15^ and Lu *et al*.^36^. Interestingly, c‒PTIO and l‒NAME application arrested the effect of Ca^2+^ and NO in As‒stressed seedlings, which was further confirmed by *in*‒*vivo* staining for SOR, H_2_O_2_, lipid peroxidation and injury of plasma membrane integrity in leaf tissues (Figs. 2a,b). To overcome the deleterious effect of As, the activities of enzymatic antioxidants: SOD and CAT were found to increase, which were further enhanced upon CaCl_2_ and SNP addition and more importantly, still an increment in the enzyme activities was noticed after c‒PTIO, l‒NAME, EGTA and LaCl_3_ treatment (Figs. 5a,b). The increased SOD and CAT activities, which established the frontline enzymatic network that dismutate O_2_˙^−^ into H_2_O_2_ consecutively into H_2_O, speeded the reduction in ROS accumulation but it was not sufficient to overcome the massive As‒induced c‒PTIO, l‒NAME, EGTA and LaCl_3_ mediated ROS accumulation; therefore higher ROS accumulation were still noticed (Figs. 2a,b) under these conditions.

### Ca^2+^ and NO recover As‒induced losses of ascorbate and glutathione contents and maintain redox status

Ascorbate and glutathione are the important redox buffering agents, therefore were analyzed in the present study to know the redox status of the cell. The current study showed that, As seriously impaired the ROS detoxification process by reducing the contents of AsA and GSH along with their redox states: AsA/DHA and GSH/GSSG ratios (Table 2). Upon addition of NO chelator (c‒PTIO) and inhibitor (l‒NAME), further reduction in the contents of AsA and GSH and ratios of AsA/DHA and GSH/GSSG was reported even in presence of Ca^2+^ (Table 2). Moreover, the activity of APX, which reduces H_2_O_2_ to DHA on the expense of AsA, was increased under similar conditions (Fig. 5c) indicating that although APX activity was efficient for H_2_O_2_ detoxification; however, this was not enough to counteract H_2_O_2_ induced damage, which is obvious from the results of MDA content and electrolyte leakage (Fig. 2). In contrast to this, DHAR activity which recycled DHA into AsA in presence of GSH, was found to decrease suggesting the insufficient regeneration of AsA from DHA; therefore much lower AsA content was noticed under As + Ca + EGTA + LaCl_3_ and As + Ca + c‒PTIO + _L_‒NAME treatment and obviously, low AsA/DHA ratio was found (Table 2). The DHAR enzyme is susceptible to high H_2_O_2_ concentration^37^; therefore upon addition of NO chelator/synthesis inhibitor, a remarkable inhibition in DHAR activity was noticed that might have altered the rate of AsA‒GSH cycle, which is apparent from decreased AsA/DHA ratio (Table 2). Calcium and NO, on the other hand restored and up‒regulated DHAR activity to maintain the higher level of AsA; therefore higher AsA/DHA ratio was noticed (Table 2), as was also argued by Ahmad *et al*.^13^. Further, during the conversion of DHA into AsA, two molecules of GSH by donating electron converted into GSSG, which in‒turn re‒reduced into GSH by glutathione reductase (GR) enzyme^38^. In the present investigation, a significant reduction in the content of GSH and subsequent increment in GSSG was found upon As treatment, and effect was more intense under NO scavenger and synthesis inhibitor, thereby causing a severe reduction in GSH/GSSG ratio (Table 2). The decrease in GSH content might either be due to decrease in GSH recycling rate or increase in its degradation rate, as excessive GSH needed during DHA to AsA conversion^38^. Interestingly, GR activity was increased upon c‒PTIO and l‒NAME treatment (Fig. 5e), which suggests that it was not sufficient to manage the huge GSH consuming effect of As, such as GSH conjugation for GST and PCs synthesis (As‒PCs complex); therefore low GSH/GSSG ratio was obtained (Table 2). Contrastingly, Ca^2+^ and NO improved the GSH pool by speeding up the rate of GSH recycling from GSSG, which is evident by increased GR activity (Fig. 5e), thus greater GSH/GSSG ratio was obtained to encounter As toxicity, as also suggested by Ahmad *et al*.^13^ and Praveen and Gupta^4^.

**Table 2:** Effect of CaCl_2_ and SNP on the contents of non‒enzymatic antioxidants: ascorbate (AsA: reduced and DHA: dehydroascorbate), glutathione (GSH: reduced and GSSG: oxidized) and their redox status (AsA/DHA and GSH/GSSG) and content of thiol compounds (cysteine: Cys, non‒protein thiols: NPTs and phytochelatins: PCs) of As‒challenged *Brassica* seedlings subjected to different modulators.

Treatments	Contents (nmol g^—1^ FW)				Redox ratios		Content of thiol compounds (nmol g^—1^ FW)		
	AsA	DHA	GSH	GSSG	AsA/DHA	GSH/GSSG	Cys	NPTs	PCs
Control	1455 ± 25^a^	106.5 ± 1.8^h^	585.5 ± 9.8^a^	46.74 ± 0.78^i^	13.66 ± 0.22^a^	12.52 ± 0.21^a^	62.15 ± 1.04^g^	761.1 ± 12.7^i^	128.8 ± 2.2^g^
As	1142 ± 15f^g^	189.1 ± 2.5^cd^	487.7 ± 6.5^cd^	82.01 ± 1.09^d^	6.04 ± 0.08^fg^	5.94 ± 0.07^g^	107.67 ± 1.43^b^	867.2 ± 11.5^h^	297.5 ± 3.9^f^
As + Ca	1408 ± 20^ab^	127.1 ± 1.8^g^	566.9 ± 8.2^a^	54.89 ± 0.79^h^	11.08 ± 0.15^b^	10.32 ± 0.14^b^	71.38 ± 1.03^f^	982.8 ± 14.2^fg^	361.1 ± 5.2^e^
As + Ca + EGTA	1104 ± 15^fg^	198.2 ± 2.7^bc^	471.0 ± 6.5^de^	89.71 ± 1.24^c^	5.57 ± 0.07^gh^	5.25 ± 0.07^h^	113.78 ± 1.57^b^	1121.0 ± 15.5^bc^	560.4 ± 7.8^b^
As + Ca + EGTA + LaCl_3_	1049 ± 17^g^	241.1 ± 3.9^a^	442.5 ± 7.1^e^	105.88 ± 1.71^a^	4.34 ± 0.07^i^	4.17 ± 0.06^i^	131.89 ± 2.13^a^	1191.0 ± 19.3^a^	642.6 ± 10.4^a^
As + Ca + c‒PTIO	1219 ± 18^de^	171.8 ± 2.6^ef^	512.6 ± 7.7^bc^	68.91 ± 1.03^ef^	7.09 ± 0.10^e^	7.44 ± 0.10^e^	95.73 ± 1.43^c^	1062.2 ± 15.9^de^	480.6 ± 7.2^d^
As + Ca + l‒NAME	1270 ± 21^cd^	158.7 ± 2.7^f^	519.4 ± 8.7^bc^	63.67 ± 1.06^fg^	8.00 ± 0.13^d^	8.15 ± 0.13^d^	86.89 ± 1.45^d^	1037.1 ± 17.4^ef^	454.0 ± 7.6^d^
As + Ca + c‒PTIO + l‒NAME	1063 ± 18^g^	208.9 ± 3.5^b^	455.4 ± 7.6^de^	97.16 ± 1.62^b^	5.09 ± 0.08^h^	4.68 ± 1.07^hi^	126.83 ± 2.12^a^	1143.0 ± 19.1^ab^	590.5 ± 9.9^b^
As + SNP	1371 ± 20^ab^	139.2 ± 2.0^g^	549.0 ± 7.9^ab^	61.56 ± 0.88^g^	9.84 ± 0.14^c^	8.92 ± 0.12^c^	78.53 ± 1.13^ef^	994.4 ± 14.4^fg^	383.8 ± 5.5^a^
As + SNP + c‒PTIO	1169 ± 15^ef^	184.4 ± 2.3^de^	493.9 ± 6.3^cd^	74.48 ± 0.94^e^	6.34 ± 0.08^f^	6.63 ± 0.08^f^	98.90 ± 1.25^c^	1087.1 ± 13.8^cd^	518.6 ± 6.6^c^
As + SNP + l‒NAME	1358 ± 22^bc^	134.1 ± 2.2^g^	564.4 ± 9.1^a^	58.07 ± 0.93^gh^	10.12 ± 0.16^c^	9.72 ± 0.15^b^	81.23 ± 1.31^de^	944.8 ± 15.3^gh^	322.3 ± 5.2^f^

Data signifies the mean ± standard error of five replicates. Values within same column followed by different subscripts are significantly different at *p *< *0.05* level according to Tukey test.

### Ca^2+^ and NO rescue As‒induced damage by up‒regulating synthesis of thiol compounds

The thiol compounds: Cys, NPTs and PCs act as first barrier against As toxicity thereby reducing the injurious effect to plants^2,4^. In the present investigation, the content of Cys, NPTs and PCs were found to increase under As stress and further enhancement was noticed when EGTA + LaCl_3_ or c‒PTIO + _L_‒NAME were supplemented to As + Ca stressed seedlings (Table 2), which might be due to their demand for Fe–S cluster of photosynthetic apparatus^39^, protein synthesis, stabilizing tertiary structures of protein, synthesis of GSH, hydroxymethyl‒PCs and other low molecular weight compounds^3,36^. Moreover, high PCs content demands more GSH to counteract the stressed situation and also higher As accumulation stimulates NPTs for scavenging, by using available GSH pool; thus lower GSH content was obtained upon EGTA + LaCl_3_ or c‒PTIO + _L_‒NAME supplementation to As + Ca treated seedlings (Table 2). Additionally, CaCl_2_ and SNP treatment to As‒stressed test seedlings also showed an improvement in the contents of thiol compounds thereby justifying their role in reducing As toxicity by promoting peptides and proteins to chelate metalloid, which is corroborated with earlier findings of Lu *et al*.^36^ and Praveen and Gupta^4^ in *Amaranthus hypochondriacus* and *Oryza sativa* seedlings, respectively.

Table S2 shows the correlation between treatments and tested parameters in the *B. juncea* L. seedlings. All the treatments affected all the tested parameters significantly. The results clearly showed that arsenic negatively affected the growth and other growth regulating parameters, while Ca and SNP showed positive correlation with growth. Further, addition of EGTA and LaCl_3_ to As + Ca; while c‒PTIO and l‒NAME to As + SNP treated seedlings significantly declined the growth (as depicted by negative correlation). Further when c‒PTIO and l‒NAME individually as well as in combination were supplemented to As + Ca treated seedlings, more negative values for pearson correlation were observed thereby signifying that NO plays crucial role in Ca^2+^ mediated signalling.

## Materials and methods

### Experimental plant, growth conditions and treatments

Healthy seeds of *Brassica juncea* were surface sterilized with 5% (v/v) sodium hypochlorite (NaOCl) for 5 min, rinsed with distilled water (DW) and left overnight in dark for 48 h by wrapping them in a wet muslin cloth. The germinated seeds were sown in plastic cups having acid sterilized sand and kept in darkness for two days at 25 ± 1 °C. Seedlings were then transferred and allowed to grow in plant growth chamber (CDR model GRW‒300 DGe, Athens, Greece) having photosynthetically active radiation (PAR) of 150 μmol photons m^−2^ s^−1^ with 16:8 h day‒night regime and 65–70% relative humidity at 22 ± 1 °C. Seedlings were irrigated with 50% Hoagland and Arnon^40^ solution on alternate days. After 25 days, seedlings were uprooted and acclimatized in 50% Hoagland solution for 24 h. After that, three healthy and uniform sized seedlings were transferred in each plastic cup having 50 ml of Hoagland solution with or without different combinations of donor, scavenger and inhibitors (doses of As, Ca and NO were selected on the basis of screening experiments; the experimental plants showing phenotypic variation as per the treatments have been shown in Supplementary Fig. S1). All the chemicals were prepared in 50% Hoagland solution. In hydroponic system, following combinations were made: (i) control (nutrient solution alone), (ii) As 50 µM, (iii) As + Ca, (iv) As + Ca + EGTA, (v) As + Ca + EGTA + LaCl_3_, (vi) As + Ca + c‒PTIO, (vii) As + Ca + l‒NAME, (viii) As + Ca + l‒NAME + c‒PTIO, (ix) As + SNP, (x) As + SNP + c‒PTIO and (xi) As + SNP + l‒NAME. Sodium arsenate (Na_2_HAsO_4_.7H_2_O: a source of As; 50 µM), calcium chloride (CaCl_2_: a Ca^2+^ donor; 12 mM), sodium nitroprusside (SNP: a NO donor; 100 µM), ethylene glycol‒bis(2‒aminoethylether)‒*N,N,N*′*,N*′‒tetraacetic acid (EGTA: a Ca^2+^ scavenger; 0.10 mM), 2‒4‒carboxyphenyl‒*4,4,5,5*‒tetramethylimidazoline‒1‒oxyl‒3‒oxide (c‒PTIO: a NO scavenger; 0.10 mM), lanthanum chloride (LaCl_3_: a plasma membrane Ca^2+^ channel blocker; 0.10 mM) and N^ω^‒nitro‒L‒arginine methyl ester hydrochloride (l‒NAME: a NO synthase inhibitor; 0.10 mM) were used as metal stress, donor, scavenger and inhibitors. Each treatment was performed in five sets and were transferred in growth chamber under similar condition as mentioned above. The seedlings were aerated regularly with air bubbler to avoid hypoxia condition and were harvested after four days of the treatments to examine the Ca^2+^ and NO‒induced mechanisms in modulating As‒induced responses.

### Growth analysis

After four days of the treatments, growth of *Brassica* seedlings was analyzed by measuring fresh weight (FW), root length (RL) and shoot length (SL). The FW of the seedling was recorded by single pan digital balance (Model CA 223, Contech, India), while RL and SL were recorded by meter scale.

### Estimation of As content

For the estimation of As content, 100 mg dried plant samples from each treatment were digested in tri‒acid mixture (HClO_4_:H_2_SO_4_:HNO_3_::1:1:5 ratio, v/v) at 80 °C according to the method of Allen *et al*.^41^. Arsenic content in digested sample was estimated by atomic absorption spectrometer (AAS, iCE 3000 series, model–3500, Thermo Scientific, UK).

### Estimation of photosynthetic pigment contents

After extracting 20 mg fresh leaves in chilled acetone (80%), absorbance of the supernatants was recorded at 470, 646 and 663 nm to determine the Chls and carotenoid contents according to the formulas suggested by Lichtenthaler^42^.

### Assay of photosynthesis and PS II photochemistry (polyphasic fast chlorophyll a fluorescence induction and JIP‒kinetics)

#### Photosynthesis

The rate of photosynthetic oxygen yield in leaves of test seedlings was measured using Clark type oxygen electrode (Digital Oxygen System, Model‒10, Rank Brothers, UK) in terms of oxygen evolution in presence of light as suggested by Kurra‒Hotta *et al*.^43^.

#### Polyphasic fast chlorophyll a fluorescence induction and JIP‒kinetics

To check the performance of photosynthesis, Chl *a* fluorescence measurements were carried out in 30 min dark‒adapted leaves using leaf fluorometer (FluorPen FP 100, Photon System Instrument, Czech Republic). The analysis of OJIP transient took into consideration by measuring the fluorescence values at 50 μs (F_O_, step O), 2 ms (F_2ms_, step J), 30 ms (F_30ms_, step I) and maximal level (F_M_, step P). The shape of OJIP rise shows the complexity of reduction kinetic of PS II (acceptor side). The O‒J favours photochemical reduction of Q_A_ (primary electron acceptor), J‒I agrees with the Q_B_ quenching mechanism or complete closure of PS II reaction centre (RC) and I‒P favours reduction of pool and size of final electron acceptor of PS I^22^.

The following biophysical parameters deduced from OJIP transient curves were calculated: (**1). Technical/absolute parameters:** (i) initial fluorescence (*F*_*o*_), (ii) maximum fluorescence (*F*_*m*_), (iii) variable fluorescence (*F*_v_; fraction of total number of closed RCs), (iv) number of Q_A_ redox turnover until *F*_*m*_ is reached (*N*) and (v) area above the fluorescence induction curve between *F*_*o*_ and *F*_*m*_, reflecting the size of plastoquinone (PQ) pool (*Area*); (**2). Quantum yields and efficiencies/probabilities of PS II:** (i) quantum yield of primary photochemistry (*ФP*_*o*_ or *Phi_P*_*o*_), (ii) yield of electron transport per trapped exciton (the probability that a trapped exciton moves an electron into the electron transport chain beyond Q_A_^−^; *Ψ*_*o*_ or *Psi_*_*o*_), (iii) quantum yield of electron transport (*ФE*_*o*_ or *Phi_E*_*o*_) and (iv) the probability that an absorbed photon is dissipated (*ФD*_*o*_); (**3). Specific energy fluxes or activities per RC (per Q**_**A**_ **reducing PS II RC):** (i) total absorption by PS II antenna Chls divided by the number of active (in sense of Q_A_ reduction) RC (*ABS/RC;* refers average antenna size), (ii) trapped energy flux per active RC [refers only to active (Q_A_‒Q_A_^−^) RCs; *TR*_*o*_*/RC*], (iii) electron transport flux from Q_A_^−^ to plastoquinone (PQ) per active RC (*ET*_*o*_*/RC*), (iv) total dissipation to the amount of active RCs (*DI*_*o*_*/RC*) and (v) energy necessary for the closure of all the RCs (*S*_*m*_); (**4). Structural and functional heterogeneity of PS II:** (i) **antenna heterogeneity** is associated with Q_A_ reduction in relation to electron flow to PQ pool or (ii) **reducing side** (functioning of reduction side *i.e*. Q_B_^−^ reducing and non*—*reducing centres): (a) efficiency of water splitting complex (*F*_*o*_*/F*_*v*_), (b) the rate of oxidation/reduction of PQ (*F*_*o*_*/F*_*m*_), (c) size and number of active photosynthetic reaction centres (*F*_*v*_*/F*_*o*_) and the ratio of fluorescence yields for open and closed states (*F*_*m*_*/F*_*o*_); (**5). Overall performance index** (*PI*_*ABS*_; the potential for energy conservation from photons absorbed by PS II to the reduction of intersystem electron acceptors) following the method of Strasser *et al*.^20^ and kalaji *et al*.^27^.

### Estimation of reactive oxygen species (ROS: SOR and H_2_O_2_) and indices (MDA equivalents content and electrolyte leakage) of damage

#### Biochemical analysis

The estimation of ROS: superoxide radical (SOR: O_2_˙^−^) and hydrogen peroxide (H_2_O_2_) contents were adopted from Elstner and Heupel^44^ and Velikova *et al*.^45^ and the amount was calculated with the help of standard curve of NaNO_2_ and H_2_O_2_, respectively. The estimation of indices of damage: lipid peroxidation (measured in terms of MDA equivalents content) and loss of membrane integrity (measured in terms of electrolyte leakage) in leaf tissues were adopted from Heath and Packer^46^ and Gong *et al*.^47^, respectively.

#### Histochemical detection

To perform the histochemical detection for SOR, H_2_O_2_, lipid peroxidation and loss of membrane integrity, nitro blue tetrazolium (NBT; 0.1%), 3,3′‒diaminobenzidine (DAB; 1%), Schiff’s reagent and Evan’s blue tests were carried out as suggested by Frahry and Schopfer^48^, Thordal‒Christensen *et al*.^49^, Pompella *et al*.^50^ and Yamamoto *et al*.^51^, respectively. After staining, leaves were bleached with boiling ethanol and photographed.

### Estimation of activities of enzymatic antioxidants

The extraction and estimation of superoxide dismutase (SOD; EC 1.15.1.1) activity in presence of riboflavin and methionine was adopted from Giannopolitis and Ries^52^, which is mainly based on photoreduction of NBT. Catalase (CAT; EC 1.11.3.6) activity was assayed in presence of H_2_O_2_ as suggested by Aebi^53^ using an extinction coefficient (ϵ) 39.4 mM^−1^ cm^−1^. Ascorbate peroxidase (APX; EC 1.11.1.11) activity was assayed in presence of ascorbate and H_2_O_2_ as suggested by Nakano and Asada^54^ using (ϵ) of 2.8 mM^−1^ cm^−1^. Estimation of dehydroascorbate reductase (DHAR; EC 2.5.1.18) activity is based on the reduction of dehydroascorbate (DHA) into reduced ascorbate (AsA) as suggested by Nakano and Asada^54^ and the activity was determined using (ϵ) of 7.0 mM^−1^ cm^−1^. The assay of glutathione reductase (GR; EC 1.6.4.2) activity is mainly based on oxidation of NADPH in presence of oxidized glutathione (GSSG) using (ϵ) of 6.2 mM^−1^ cm^−1^ as suggested by Schaedle and Bassham^55^.

One unit (U) of activity of SOD is the amount of SOD required to inhibit 50% NBT, CAT (U) is equivalent to 1 nmol H_2_O_2_ dissociated min^−1^, APX (U) is 1 nmol ascorbate oxidized min^−1^, DHAR (U) is 1 nmol DHA reduced min^−1^ and GR (U) is defined as 1 nmol NADPH oxidized min^−1^.

### Estimation of non‒enzymatic antioxidants: ascorbate, glutathione and their redox status

The estimation of contents of ascorbate: reduced (AsA) and oxidised (dehydroascorbate: DHA) was carried out in acidic solution, which is mainly based on Fe^3+^ reduction into Fe^2+^ following the method of Gossett *et al*.^56^. The estimation of glutathione: reduced (GSH) and oxidized (GSSG) contents were based on the sequential oxidation of GSH by 5,5′‒dithiobis‒2‒nitrobenzoic acid (DTNB) into trinitrobenzoic acid (TNB) as suggested by Brehe and Burch^57^.

### Estimation of thiol compounds: cysteine, non‒protein thiols and phytochelatins

Estimation of cysteine (Cys) content was done in presence of glacial acetic acid (GAA), acid ninhydrin and toluene following the method of Gaitonde^58^. The content of non‒protein thiols (NPTs) was measured according to Ellman^59^ in presence of Ellman’s reagent. The amount of total phytochelatins (PCs) was calculated using the formula: total PCs = NPTs‒total GSH^60^.

### Estimation of nitric oxide (NO) content

The NO content was determined using the method described by Zhou *et al*.^61^. The 500 mg fresh leaf tissues were homogenized in 50 mM acetic acid buffer (pH 3.6) containing zinc diacetate (4%) and centrifuged for 15 min at 4 °C. The absorbance of reaction mixture containing charcoal and 1 ml of Greiss reagent was monitoring at 540 nm and NO content in the mixture was calculated with the help of standard curve of NaNO_2_.

### Statistical analysis

The experiments were performed in quintuplicates and the results displayed in figures and tables are the means ± standard error of the average values obtained from quintuplicates (n = 5) of individual experiment to check the reproducibility of result. The results were statistically analyzed by one‒way analysis of variance (ANOVA) using software ‘SPSS 16.0. Tukey alpha test was performed for the mean separation for significant differences among the treatments at *p* < *0.05* significance level. Pearson correlation coefficient (r) test was also applied to test the significance of treatments.

## Conclusion

From the present study, it can be concluded that As inhibits growth of *Brassica* seedlings; Ca^2+^ and NO on the other hand recovered the growth and growth related parameters. However, with the addition of Ca^2+^ chelator (EGTA), plasma membrane Ca^2+^ channel blocker (LaCl_3_) as well as NO chelator (c‒PTIO) and synthetase inhibitor (_L‒_NAME), the improvement in growth caused by Ca^2+^ and NO was further arrested, which suggests that both are involve in signalling network. When NO chelator and synthase inhibitor were added to As‒stressed CaCl_2_ supplemented seedlings, a steep decline in growth promoting processes: photosynthetic activity, Chl *a* fluorescence, redox status (AsA/DHA and GSH/GSSG) of the cell was noticed even in presence of Ca^2+^, thereby signifying that physiological and biochemical attributes of *Brassica* seedlings are mostly regulated by intensive Ca^2+^ mediated NO signalling.

## Supplementary information

Supplementary Information.
