# Publisher Correction: Interplay of Calcium and Nitric Oxide in improvement of Growth and Arsenic-induced Toxicity in Mustard Seedlings

**DOI:** 10.1038/s41598-020-69172-y

**Published:** 2020-07-21

**Authors:** Rachana Singh, Parul Parihar, Sheo Mohan Prasad

**Affiliations:** 1 Ranjan Plant Physiology and Biochemistry Laboratory, Department of Botany, University of Allahabad, Allahabad, U.P. India; 2 School of Bioengineering and Biosciences, Lovely Professional University, Phagwara, India

Correction to: *Scientific Reports* 10.1038/s41598-020-62831-0, published online 23 April 2020

In the original version of this Article, Rachana Singh was incorrectly listed as the corresponding author. The correct corresponding author for this Article is Sheo Mohan Prasad. Correspondence and request for materials should be addressed to profsmprasad@gmail.com. This error has now been corrected in the HTML and PDF of the article.

