# Supplementary material for: Interplay of Calcium and Nitric Oxide in improvement of Growth and Arsenic-induced Toxicity in Mustard Seedlings
Source: Sci Rep. 2020 Apr 23;10:6900. doi: 10.1038/s41598-020-62831-0 (PMC7181649; doi:10.1038/s41598-020-62831-0)
Supplement: Supplementary file 1 — Supplementary Information. [file 41598_2020_62831_MOESM1_ESM.docx]

**Title:** Interplay of Calcium and Nitric Oxide in improvement of Growth and Arsenic-induced Toxicity in Mustard Seedlings

**Authors:** Rachana Singh, Parul Parihar, Sheo Mohan Prasad

**Figure S1.** Impact of As, Ca and different NO modulators [(I) Control, (II) As, (III) As+Ca, (IV) As+Ca+EGTA, (V) As+Ca+EGTA+LaCl_3_, (VI) As+Ca+c‒PTIO, (VII) As+Ca+_L_‒NAME, (VIII) As+Ca+c‒PTIO+_L_‒NAME, (IX) As+SNP, (X) As+SNP+c‒PTIO and (XI) As+SNP+_L_‒NAME] on the phenotype of *Brassica juncea* L. seedlings.

**Table S2**. Pearson correlation coefficient (*r*) value showing effect of different modulators, scavengers and inhibitors on growth and growth regulating process in *Brassica juncea* L seedlings.

| **Treatments** | **FW** | **RL** | | **SL** | | **Chl *a*** | | **Chl *b*** | | **Car** | | **Photosynthesis** | | **As accumulation** | | | **SOR** |
| --- | --- | --- | --- | --- | --- | --- | --- | --- | --- | --- | --- | --- | --- | --- | --- | --- | --- |
| **As** | ***r***= -0.991^***^  **P***<*0.001 | ***r***= -0.993^***^  **P***<*0.001 | | ***r***= -0.991^***^  **P***<*0.001 | | ***r***= -0.958^**^  **P***<*0.003 | | ***r***= -0.990^***^  **P***<*0.001 | | ***r***= -0.935^**^  **P***<*0.006 | | ***r***= -0.996^***^  **P***<*0.001 | | ***r***=1.000^***^  **P***<*0.001 | | | ***r***=0.998^***^  **P***<*0.001 |
|  |  |  | |  | |  | |  | |  | |  | |  | | |  |
| **As+Ca** | ***r***= -0.914^*^  **P***<*0.011 | ***r***= -0.948^**^  **P***<*0.004 | | ***r***= -0.875^*^  **P***<*0.022 | | ***r***= -0.871^*^  **P***<*0.024 | | ***r***= -0.969^***^  **P***<*0.001 | | ***r***= -0.874^*^  **P***<*0.023 | | ***r***= -0.811^*^  **P***<*0.050 | | ***r***= 1.000^***^  **P***<*0.001 | | | ***r***= 0.980^***^  **P***<*0.001 |
|  |  |  | |  | |  | |  | |  | |  | |  | | |  |
| **As+Ca+EGTA** | ***r***= -0.992^***^  **P***<*0.001 | ***r***= -0.991^***^  **P***<*0.001 | | ***r***= -0.992^***^  **P***<*0.001 | | ***r***= -0.963^**^  **P***<*0.002 | | ***r***= -0.990^***^  **P***<*0.001 | | ***r***= -0.965^**^  **P***<*0.002 | | ***r***= -0.997^***^  **P***<*0.001 | | ***r***= 1.000^***^  **P***<*0.001 | | | ***r***= 0.998^***^  **P***<*0.001 |
|  |  |  | |  | |  | |  | |  | |  | |  | | |  |
| **As+Ca+EGTA+LaCl_3_** | ***r***= -0.981^***^  **P***<*0.001 | ***r***= -0.984^***^  **P***<*0.001 | | ***r***= -0.986^***^  **P***<*0.001 | | ***r***= -0.928^**^  **P***<*0.008 | | ***r***= -0.982^***^  **P***<*0.001 | | ***r***= -0.939^**^  **P***<*0.005 | | ***r***= -0.991^***^  **P***<*0.001 | | ***r***= 1.000^***^  **P***<*0.001 | | | ***r***= 0.995^***^  **P***<*0.001 |
|  |  |  | |  | |  | |  | |  | |  | |  | | |  |
| **As+Ca+c‒PTIO** | ***r***= -0.994^***^  **P***<*0.001 | ***r***= -0.994^***^  **P***<*0.001 | | ***r***= -0.992^***^  **P***<*0.001 | | ***r***= -0.979^***^  **P***<*0.001 | | ***r***= -0.991^***^  **P***<*0.001 | | ***r***= -0.974^***^  **P***<*0.001 | | ***r***= -0.998^***^  **P***<*0.001 | | ***r***= 0.999^***^  **P***<*0.001 | | | ***r***= 0.998^***^  **P***<*0.001 |
|  |  |  | |  | |  | |  | |  | |  | |  | | |  |
| **As+Ca+ʟ‒NAME** | ***r***= -0.975^***^  **P***<*0.001 | ***r***= -0.967^**^  **P***<*0.002 | | ***r***= -0.977^***^  **P***<*0.001 | | ***r***= -0.902^*^  **P***<*0.014 | | ***r***= -0.974^***^  **P***<*0.001 | | ***r***= -0.936^**^  **P***<*0.006 | | ***r***= -0.987^***^  **P***<*0.001 | | ***r***= 0.999^***^  **P***<*0.001 | | | ***r***= 0.992^***^  **P***<*0.001 |
|  |  |  | |  | |  | |  | |  | |  | |  | | |  |
| **As+Ca+c-PTIO+ʟ‒NAME** | ***r***= -0.952^**^  **P***<*0.003 | ***r***= -0.958^**^  **P***<*0.003 | | ***r***= -0.945^**^  **P***<*0.004 | | ***r***= -0.904^*^  **P***<*0.013 | | ***r***= -0.966^**^  **P***<*0.002 | | ***r***= -0.868^*^  **P***<*0.025 | | ***r***= -0.978^***^  **P***<*0.001 | | ***r***= 0.999^***^  **P***<*0.001 | | | ***r***= 0.990^***^  **P***<*0.001 |
|  |  |  | |  | |  | |  | |  | |  | |  | | |  |
| **As+SNP** | ***r***= -0.990^***^  **P***<*0.001 | ***r***= -0.993^***^  **P***<*0.001 | | ***r***= -0.993^***^  **P***<*0.001 | | ***r***= -0.948^**^  **P***<*0.004 | | ***r***= -0.960^**^  **P***<*0.002 | | ***r***= -0.968^**^  **P***<*0.002 | | ***r***= -0.994^***^  **P***<*0.001 | | ***r***= 1.000^***^  **P***<*0.001 | | | ***r***= 0.998^***^  **P***<*0.001 |
|  |  |  | |  | |  | |  | |  | |  | |  | | |  |
| **As+SNP+c‒PTIO** | ***r***= -0.950^**^  **P***<*0.004 | ***r***= -0.955^**^  **P***<*0.003 | | ***r***= -0.908^*^  **P***<*0.012 | | ***r***= -0.891^*^  **P***<*0.017 | | ***r***= -0.970^***^  **P***<*0.001 | | ***r***= -0.869^*^  **P***<*0.025 | | ***r***= -0.963^**^  **P***<*0.002 | | ***r***= 1.000^***^  **P***<*0.001 | | | ***r***= 0.987^***^  **P***<*0.001 |
|  |  |  | |  | |  | |  | |  | |  | |  | | |  |
| **As+SNP+ʟ‒NAME** | ***r***= -0.993^***^  **P***<*0.001 | ***r***= -0.993^***^  **P***<*0.001 | | ***r***= -0.992^***^  **P***<*0.001 | | ***r***= -0.970^***^  **P***<*0.001 | | ***r***= -0.993^***^  **P***<*0.001 | | ***r***= -0.977^***^  **P***<*0.001 | | ***r***= -0.997^***^  **P***<*0.001 | | ***r***= 0.999^***^  **P***<*0.001 | | | ***r***= 0.998^***^  **P***<*0.001 |
| **Treatments** | **H_2_O_2_** | | **MDA** | | **EL** | | **SOD** | | **CAT** | | **APX** | | **DHAR** | | **GR** | **Cysteine** | |
| **As** | ***r***= 0.996^***^  **P***<*0.001 | | ***r***= 0.989^***^  **P***<*0.001 | | ***r***= 0.990^***^  **P***<*0.001 | | ***r***= 0.997^***^  **P***<*0.001 | | ***r***= 0.999^***^  **P***<*0.001 | | ***r***= 0.994^***^  **P***<*0.001 | | ***r***= 1.000^***^  **P***<*0.001 | | ***r***= 0.999^***^  **P***<*0.001 | ***r***= 0.997^***^  **P***<*0.001 | |
|  |  | |  | |  | |  | |  | |  | |  | |  |  | |
| **As+Ca** | ***r***= 0.974^***^  **P***<*0.001 | | ***r***= 0.899^*^  **P***<*0.015 | | ***r***= 0.944^**^  **P***<*0.005 | | ***r***= 0.963^**^  **P***<*0.002 | | ***r***= 0.995^***^  **P***<*0.001 | | ***r***= -0.873^*^  **P***<*0.023 | | ***r***= -0.826^*^  **P***<*0.043 | | ***r***= 0.979^***^  **P***<*0.001 | ***r***= 0.953^**^  **P***<*0.003 | |
|  |  | |  | |  | |  | |  | |  | |  | |  |  | |
| **As+Ca+EGTA** | ***r***= 0.995^***^  **P***<*0.001 | | ***r***= 0.990^***^  **P***<*0.001 | | ***r***= 0.990^***^  **P***<*0.001 | | ***r***= 0.997^***^  **P***<*0.001 | | ***r***= 0.999^***^  **P***<*0.001 | | ***r***= 0.995^***^  **P***<*0.001 | | ***r***= -0.996^***^  **P***<*0.001 | | ***r***= 0.999^***^  **P***<*0.001 | ***r***= 0.997^***^  **P***<*0.001 | |
|  |  | |  | |  | |  | |  | |  | |  | |  |  | |
| **As+Ca+EGTA+LaCl_3_** | ***r***= 0.993^***^  **P***<*0.001 | | ***r***= 0.981^***^  **P***<*0.001 | | ***r***= 0.985^***^  **P***<*0.001 | | ***r***= 0.993^***^  **P***<*0.001 | | ***r***= 0.998^***^  **P***<*0.001 | | ***r***= 0.990^***^  **P***<*0.001 | | ***r***= -0.990^***^  **P***<*0.001 | | ***r***= 0.998^***^  **P***<*0.001 | ***r***= 0.994^***^  **P***<*0.001 | |
|  |  | |  | |  | |  | |  | |  | |  | |  |  | |
| **As+Ca+c‒PTIO** | ***r***= 0.996^***^  **P***<*0.001 | | ***r***= 0.991^***^  **P***<*0.001 | | ***r***= 0.998^***^  **P***<*0.001 | | ***r***= 0.999^***^  **P***<*0.001 | | ***r***= 0.996^***^  **P***<*0.001 | | ***r***= -0.998^***^  **P***<*0.001 | | ***r***= 0.999^***^  **P***<*0.001 | | ***r***= 0.998^***^  **P***<*0.001 | ***r***= 0.998^***^  **P***<*0.001 | |
|  |  | |  | |  | |  | |  | |  | |  | |  |  | |
| **As+Ca+ʟ‒NAME** | ***r***= 0.991^***^  **P***<*0.001 | | ***r***= 0.973^***^  **P***<*0.001 | | ***r***= 0.980^***^  **P***<*0.001 | | ***r***= 0.990^***^  **P***<*0.001 | | ***r***= 0.997^***^  **P***<*0.001 | | ***r***= 0.985^***^  **P***<*0.001 | | ***r***= -0.972^***^  **P***<*0.001 | | ***r***= 0.997^***^  **P***<*0.001 | ***r***= 0.990^***^  **P***<*0.001 | |
|  |  | |  | |  | |  | |  | |  | |  | |  |  | |
| **As+Ca+c-PTIO+ʟ‒NAME** | ***r***= 0.987^***^  **P***<*0.001 | | ***r***= 0.969^***^  **P***<*0.001 | | ***r***= 0.976^***^  **P***<*0.001 | | ***r***= 0.956^**^  **P***<*0.003 | | ***r***= 0.996^***^  **P***<*0.001 | | ***r***= 0.873^*^  **P***<*0.023 | | ***r***= -0.957^**^  **P***<*0.003 | | ***r***= 0.997^***^  **P***<*0.001 | ***r***= 0.985^***^  **P***<*0.001 | |
|  |  | |  | |  | |  | |  | |  | |  | |  |  | |
| **As+SNP** | ***r***= 0.996^***^  **P***<*0.001 | | ***r***= 0.989^***^  **P***<*0.001 | | ***r***= 0.987^***^  **P***<*0.001 | | ***r***= 0.996^***^  **P***<*0.001 | | ***r***= 0.999^***^  **P***<*0.001 | | ***r***= 0.994^***^  **P***<*0.001 | | ***r***= -0.992^***^  **P***<*0.001 | | ***r***= 0.999^***^  **P***<*0.001 | ***r***= 0.996^***^  **P***<*0.001 | |
|  |  | |  | |  | |  | |  | |  | |  | |  |  | |
| **As+SNP+c‒PTIO** | ***r***= 0.987^***^  **P***<*0.001 | | ***r***= 0.954^**^  **P***<*0.003 | | ***r***= 0.973^***^  **P***<*0.001 | | ***r***= 0.972^***^  **P***<*0.001 | | ***r***= 0.997^***^  **P***<*0.001 | | ***r***= 0.966^**^  **P***<*0.002 | | ***r***= -0.891^*^  **P***<*0.017 | | ***r***= 0.993^***^  **P***<*0.001 | ***r***= 0.983^***^  **P***<*0.001 | |
|  |  | |  | |  | |  | |  | |  | |  | |  |  | |
| **As+SNP+ʟ‒NAME** | ***r***= 0.995^***^  **P***<*0.001 | | ***r***= 0.990^***^  **P***<*0.001 | | ***r***= 0.989^***^  **P***<*0.001 | | ***r***= 0.997^***^  **P***<*0.001 | | ***r***= 0.998^***^  **P***<*0.001 | | ***r***= 0.995^***^  **P***<*0.001 | | ***r***= -0.997^***^  **P***<*0.001 | | ***r***= 0.999^***^  **P***<*0.001 | ***r***= 0.997^***^  **P***<*0.001 | |

| **Treatments** | **NPT** | **PCs** | **AsA** | **DHA** | **AsA/DHA** | **GSH** | **GSSG** | **GSH/GSSG** |
| --- | --- | --- | --- | --- | --- | --- | --- | --- |
| **As** | ***r***= 0.951^**^  **P***<*0.003 | ***r***= 0.999^***^  **P***<*0.001 | ***r***= -0.984^***^  **P***<*0.001 | ***r***= 0.997^***^  **P***<*0.001 | ***r***= -0.998^***^  **P***<*0.001 | ***r***= -0.972^***^  **P***<*0.001 | ***r***= 0.997^***^  **P***<*0.001 | ***r***= -0.998^***^  **P***<*0.001 |
|  |  |  |  |  |  |  |  |  |
| **As+Ca** | ***r***= 0.986^***^  **P***<*0.001 | ***r***= 0.999^***^  **P***<*0.001 | ***r***= -0.593 ^ns^  **P***<*0.215 | ***r***= 0.970^***^  **P***<*0.001 | ***r***= -0.978^***^  **P***<*0.001 | ***r***= -0.590^ns^  **P***<*0.218 | ***r***= 0.965^**^  **P***<*0.002 | ***r***= -0.974^***^  **P***<*0.001 |
|  |  |  |  |  |  |  |  |  |
| **As+Ca+EGTA** | ***r***=0.994^***^  **P***<*0.001 | ***r***= 0.999^***^  **P***<*0.001 | ***r***= -0.987^***^  **P***<*0.001 | ***r***= 0.997^***^  **P***<*0.001 | ***r***= -0.998^***^  **P***<*0.001 | ***r***= -0.980^***^  **P***<*0.001 | ***r***= 0.998^***^  **P***<*0.001 | ***r***= -0.998^***^  **P***<*0.001 |
|  |  |  |  |  |  |  |  |  |
| **As+Ca+EGTA+LaCl_3_** | ***r***= 0.991^***^  **P***<*0.001 | ***r***= 0.999^***^  **P***<*0.001 | ***r***= -0.968^***^  **P***<*0.001 | ***r***= 0.995^***^  **P***<*0.001 | ***r***=-0.997^***^  **P***<*0.001 | ***r***= -0.946^**^  **P***<*0.004 | ***r***= 0.993^***^  **P***<*0.001 | ***r***= -0.996^***^  **P***<*0.001 |
|  |  |  |  |  |  |  |  |  |
| **As+Ca+c‒PTIO** | ***r***= 0.994^***^  **P***<*0.001 | ***r***= 0.999^***^  **P***<*0.001 | ***r***= -0.989^***^  **P***<*0.001 | ***r***= 0.998^***^  **P***<*0.001 | ***r***= -0.999^***^  **P***<*0.001 | ***r***= -0.986^***^  **P***<*0.001 | ***r***= 0.998^***^  **P***<*0.001 | ***r***= -0.999^***^  **P***<*0.001 |
|  |  |  |  |  |  |  |  |  |
| **As+Ca+ʟ‒NAME** | ***r***= 0.988^***^  **P***<*0.001 | ***r***= 0.999^***^  **P***<*0.001 | ***r***= -0.944^**^  **P***<*0.005 | ***r***= 0.993^***^  **P***<*0.001 | ***r***= -0.996^***^  **P***<*0.001 | ***r***= -0.930^**^  **P***<*0.007 | ***r***= 0.988^***^  **P***<*0.001 | ***r***= -0.993^***^  **P***<*0.001 |
|  |  |  |  |  |  |  |  |  |
| **As+Ca+c-PTIO+ʟ‒NAME** | ***r***= 0.977^***^  **P***<*0.001 | ***r***= 0.998^***^  **P***<*0.001 | ***r***= -0.828^*^  **P***<*0.042 | ***r***= 0.980^***^  **P***<*0.001 | ***r***= -0.988^***^  **P***<*0.001 | ***r***= -0.619 ^ns^  **P***<*0.190 | ***r***= 0.978^***^  **P***<*0.001 | ***r***= -0.983^***^  **P***<*0.001 |
|  |  |  |  |  |  |  |  |  |
| **As+SNP** | ***r***= 0.993^***^  **P***<*0.001 | ***r***= 0.999^***^  **P***<*0.001 | ***r***= -0.981^***^  **P***<*0.001 | ***r***= 0.997^***^  **P***<*0.001 | ***r***= -0.998^***^  **P***<*0.001 | ***r***= -0.969^***^  **P***<*0.001 | ***r***= 0.996^***^  **P***<*0.001 | ***r***= -0.997^***^  **P***<*0.001 |
|  |  |  |  |  |  |  |  |  |
| **As+SNP+c‒PTIO** | ***r***= 0.987^***^  **P***<*0.001 | ***r***= 0.999^***^  **P***<*0.001 | ***r***= -0.801 ^ns^  **P***<*0.055 | ***r***= 0.987^***^  **P***<*0.001 | ***r***= -0.990^***^  **P***<*0.001 | ***r***= -0.823^*^  **P***<*0.044 | ***r***= 0.987^***^  **P***<*0.001 | ***r***= -0.991^***^  **P***<*0.001 |
|  |  |  |  |  |  |  |  |  |
| **As+SNP+ʟ‒NAME** | ***r***= 0.993^***^  **P***<*0.001 | ***r***= 0.999^***^  **P***<*0.001 | ***r***= -0.988^***^  **P***<*0.001 | ***r***= 0.997^***^  **P***<*0.001 | ***r***= -0.998^***^  **P***<*0.001 | ***r***= -0.982^***^  **P***<*0.001 | ***r***= 0.997^***^  **P***<*0.001 | ***r***= -0.998^***^  **P***<*0.001 |

**Note**: Positive correlation (+) showed that values for selected parameters were increased while negative correlation (-) showed that the values were decreased with increasing concentration of arsenic. ^*^Significant at p<0.05, ^**^Significant at p<0.01, ^***^Significant at p<0.001
